# Whose job? The staffing of advance care planning support in twelve international healthcare organizations: a qualitative interview study

**DOI:** 10.1186/s12904-018-0333-1

**Published:** 2018-05-24

**Authors:** Josie Dixon, Martin Knapp

**Affiliations:** Personal Social Services Research Unit (PSSRU), London School of Economics and Political Science (LSE), Houghton Street, London, WC2A 2AE UK

**Keywords:** Advance care planning, End of life care, Healthcare workforce, Social care

## Abstract

**Background:**

ACP involving a facilitated conversation with a health or care professional is more effective than document completion alone. In policy, there is an expectation that health and care professionals will provide ACP support, commonly within their existing roles. However, the potential contributions of different professionals are outlined only broadly in policy and guidance. Research on opportunities and barriers for involving different professionals in providing ACP support, and feasible models for doing so, is currently lacking.

**Methods:**

We identified twelve healthcare organizations aiming to offer system-wide ACP support in the United States, Canada, Australia and New Zealand. In each, we conducted an average 13 in-depth interviews with senior managers, ACP leads, dedicated ACP facilitators, physicians, nurses, social workers and other clinical and non-clinical staff. Interviews were analyzed thematically using NVivo software.

**Results:**

Organizations emphasized leadership for ACP support, including strategic support from senior managers and intensive day-to-day support from ACP leads, to support staff to deliver ACP support within their existing roles. Over-reliance on dedicated facilitators was not considered sustainable or scalable. We found many professionals, from all backgrounds, providing ACP support. However, there remained barriers, particularly for facilitating ACP conversations. A significant barrier for all professionals was lack of time. Physicians sometimes had poor communication skills, misunderstood medico-legal aspects and tended to have conversations of limited scope late in the disease trajectory. However, they could also have concerns about the appropriateness of ACP conversations conducted by others. Social workers had good facilitation skills and understood legal aspects but needed more clinical support than nurses. While ACP support provided alongside and as part of other care was common, ACP conversations in this context could easily get squeezed out or become fragmented. Referrals to other professionals could be insecure. Team-based models involving a physician and a nurse or social worker were considered cost-effective and supportive of good quality care but could require some additional resource.

**Conclusions:**

Effective staffing of ACP support is likely to require intensive local leadership, attention to physician concerns while avoiding an entirely physician-led approach, some additional resource and team-based frameworks, including in evolving models of care for chronic illness and end of life.

## Background

Advance care planning (ACP) provides an opportunity for people to discuss and record their preferences, goals and decisions for future care. This allows people's wishes to be known in the event that they are unable to speak for themselves, for people and their families to be more prepared for later decision-making and for professionals to be better able to plan ahead so they can provide the most appropriate care. In the United States (US), people can complete an advance directive, which can include a refusal of treatment, a statement of general preferences and assignment of Durable Power of Attorney (DPoA) for health care or finances. Similarly, in England and Wales, people can complete an advance decision to refuse treatment (ADRT), make an advance statement setting out general preferences and assign a Lasting Power of Attorney for health and welfare or property and financial affairs. While legal arrangements and terminology vary, similar frameworks exist in comparable countries such as Canada, Australia and New Zealand. ACP has been associated with improved end of life outcomes including fewer emergency admissions, less time spent in hospital, improved symptom control, increased carer satisfaction and potentially better use of resources [1, 2]. ACP involving a facilitated conversation with a health or care professional has been found to be more effective than document completion alone [1]. In policy, there is an expectation that health and care professionals will provide ACP support within their existing roles [3–7]. ACP support includes making people aware of ACP, facilitating ACP conversations and helping people to record their preferences in ways that can be effectively shared. However, the potential contributions of different professionals are outlined only broadly in policy and guidance, while guidance for specific professional groups and national training have not, in themselves, led to widespread implementation [8, 9]. Without a clear professional lead role, those designing and implementing system-wide strategies for providing ACP support need information about the opportunities and challenges for engaging different health and care professionals and about models for incorporating ACP provision into general care that have proved feasible in practice. Existing evidence is limited. A number of ACP intervention studies have reported on implementation challenges [10], while challenges experienced by a range of professionals in delivering ACP support, including to people with specific conditions, have been explored in a small number of qualitative interview or focus group studies [8, 9, 11–13] and in one national survey of nephrology health professionals involving a non-probability sample [14]. These studies have involved various professionals including general practitioners (GPs), hospital consultants, nurses, social workers, old-age psychiatrists, ambulance staff and physiotherapists, while intervention studies have generally involved staff, commonly clinical nurse specialists, selected and prepared by researchers [10]. Our study adds to the literature by exploring staffing of ACP support from an organizational perspective, drawing on the experiences of twelve healthcare organizations with experience of implementing ACP support across their full range of services. The healthcare organizations that participated in this study are based in four countries with well-developed ACP policy; the United States (US), Canada, Australia and New Zealand. Our research thus also responds to suggestions made in the literature that lessons be identified from international experiences of delivering ACP [15, 16]. Our specific research aims were to:

- provide a descriptive overview of how ACP support is staffed in participating healthcare organizations
- identify opportunities and barriers encountered for involving different types of health and care staff in the provision of ACP support
- identify models for delivering ACP support, with a focus on how health and care staff incorporate ACP support into their existing roles
- identify key themes and ongoing challenges.

## Methods

This study is a predominantly exploratory (contextual) qualitative interview study [17] with staff in a sample of twelve international healthcare organizations, each systematically offering ACP support across all of their services. A qualitative approach was adopted to elicit provider perspectives and to explore professionals’ first-hand experiences of developing, delivering and staffing ACP support in their organizations.

### Sampling and recruitment

A two-stage purposive sampling process was adopted; sampling healthcare organizations and then staff within these organizations. For the first stage, we sought healthcare organizations with ‘well-established, system-wide ACP support provision’. ‘ACP support provision’ was defined as helping patients and members of the public find out about ACP, facilitating ACP conversations and assisting with the completion of ACP documents. ‘Well-established’ was defined as provision that had been in place for a minimum of 18 months, but longer where possible. ‘System-wide provision of ACP’ was defined as ACP support being widely and systematically offered across all relevant services and parts of the organization. We also sought diversity against a range of secondary sampling criteria, including different countries and geographies, small and large providers, rural and urban areas, a range of care settings and varied ACP materials and approaches. We estimated that approximately 10 healthcare organizations would provide a sufficient range of experiences and enable diversity against secondary sampling criteria.

Suitable healthcare organizations were identified by drawing upon our own knowledge of relevant practice and literature and through expert, network and snowball sampling (used where sample units are rare, hidden or where there is no available sample frame). While these approaches are prone to identifying sample units within closed systems, we deliberately approached experts with a wide view of international practice, attempted to create different ‘start points’, took an iterative approach and used multiple sampling strategies [18]. Experts included four members of the International Society of Advance Care Planning and End of Life Care (ACPEL), which organizes a highly-regarded biennial international conference. We additionally asked each organization we approached to identify other suitable healthcare organizations or well-placed informants. Finally, we conducted eight scoping interviews with end of life care experts in the UK, including representatives from the National Council of Palliative Care, Care England and the Association of Directors of Adult Social Services (ADASS). We were able to sample until no more cases likely to add sufficiently new information were identified (e.g. we chose not to sample further organizations using the Respecting Choices approach or allied to the National ACP Cooperative New Zealand) [19]. It is possible that there were relevant organizations outside of our networks. In particular, we may have identified organizations most active in knowledge-exchange networks, including but not limited to ACPEL. However, arguably healthcare systems in these networks may be those with the most developed ACP support. In total, we recruited 12 healthcare organizations, some of which were geographically near to each other. These exhibited a good spread against secondary sampling criteria. Table 1 describes the organizations and indicates how they were identified.

**Table 1:** Participating healthcare organizations

	Description of organization	How organization was identified
United States		
Gundersen Health	A physician-led, not-for-profit healthcare system; birthplace of Respecting Choices, an evidence-based ACP model for person-centered decision making.	Snowball sampling via Wisconsin Medical Society, and known to the authors through the literature
Dartmouth-Hitchcock	A non-profit, academic health system, providing ACP support using the Honoring Care Decisions ACP programme (based on the Respecting Choices model).	Snowball sampling via *Gundersen Health*
Wisconsin Medical Society	A physician member association supporting 32 participating health organizations to implement the Honoring Choices ACP programme (based on the Respecting Choices model).	Known to the authors through an earlier study they led into the economics of ACP
Sharp Healthcare	A not-for-profit, integrated regional health care system, providing ACP support in collaboration with the Coalition for Compassionate Care of California.	An academic expert identified through ACPEL^a^ made an introduction to a regional coalition organization that, in turn, made an onward introduction to *Sharp Healthcare*. *Sharp Healthcare*, and its Transitions program were also known to the authors through the literature
Canada		
Northern Alberta Renal Program (NARP)	Renal programme in Edmonton, Alberta, providing integrated ACP support using an approach based on Conversations Matter.	Identified directly through a clinician, academic and member of ACPEL^a^
Fraser Health	One of six publicly funded health care regions in British Columbia, providing ACP support in community, acute and residential care based on materials developed provincially and at Fraser Health Authority.	*Northern Alberta Renal Program* (NARP) made an introduction to an academic expert in Alberta who, in turn, made an onward introduction to Fraser Health
Australia		
Austin Health	A publicly-funded health service in Melbourne, providing acute, sub-acute, mental health and ambulatory services, providing ACP support using materials developed locally and as part of Advance Care Planning Australia	Identified directly through a clinician and member of ACPEL^a^
Northern Health	A publicly-funded provider of acute, sub-acute and ambulatory specialist services in Melbourne, providing ACP support using the ‘A-C-P in three steps’ approach developed within Northern Health.	Identified through snowball sampling via *Austin Health*
Barwon Health	A publicly-funded, large regional health service, providing acute, sub-acute, elderly care, community health and mental health services, with ACP support delivered across secondary and primary care using materials, including MyValues, developed in Barwon Health.	Identified through snowball sampling via *Austin Health*
Albany Health	A regional primary and secondary healthcare system, providing ACP support using forms developed by the Western Australian government and piloting systems for communication and access of ACP documents.	Identified through an academic and member of ACPEL^a^ and through a contact identified by the authors in an earlier study they led into the economics of ACP
New Zealand		
The Canterbury Initiative	A District Health Board initiative, delivering change and quality improvement initiatives across community, primary and secondary care and providing ACP support using materials developed by the Canterbury Initiative and by the National ACP Cooperative, New Zealand.	A clinician and member of ACPEL^a^ made an introduction to the National ACP Cooperative who, in turn, made an onward introduction to the Canterbury Initiative
Auckland District Health Board	A regional health authority overseeing community, primary and secondary care, providing ACP support using material developed by the National ACP Cooperative, New Zealand	Identified through a clinician and member of ACPEL^a^

^a^The International Society of Advance Care Planning and End of Life Care

Each organization was approached directly, usually by email, through an appropriate senior member of staff. This was followed with one or more telephone conversations with senior staff to explore eligibility and discuss what participation would involve. All organizations received written information about the study. Once organizational and local ethical approvals were secured we undertook the second stage of sampling.

Stage 2 involved sampling staff within each organization. The key contact person compiled a list of all key personnel with in-depth experience of developing or delivering ACP support, and a range of others with more routine experience, to include a mix of senior managers, dedicated ACP staff, physicians, nurses, social workers, volunteer staff and other clinical and non-clinical staff. These lists were then narrowed, in consultation, with a view to balancing staff with different roles and for reasons of manageability or availability.

All identified staff were sent an introductory letter including information about the study and about what participation would involve. They were informed of the voluntary nature of participation and given the opportunity to opt out of further contact by email or by post, addressed to either the key contact person in the organization or a member of the research team, as preferred. If they did not opt out, they were invited to interview. These were scheduled, for logistic reasons, by the key contact person. Participants were informed that they could withdraw at any time and were also provided contact details for an independent person responsible for research ethics at LSE if they had concerns or queries about the conduct of recruitment or interviews.

### Conduct of interviews

We conducted between 3 and 25 (average 13) interviews in each organization [17] during fieldwork visits undertaken between November 2015 and May 2017 (Table 2). Most were individual (*n* = 112) although occasionally, for practicality, group interviews were conducted (*n* = 18). Interviews ranged from 20 to 180 min. Fully informed verbal consent was obtained at the beginning of each interview. Interviews covered various topics, with an over-arching focus on resources used to deliver ACP support. Topic coverage was adapted to reflect the role and expertise of interviewees. In particular, respondents were asked to describe their own role and experiences of providing ACP support and about the staffing of ACP support more generally. All responses were thoroughly probed. Quantitative data about staffing for ACP support were enquired about but availability was limited. Interviews were audio-recorded with permission.

**Table 2:** Interviews by healthcare system and respondent role

	Gundersen	Dartmouth-Hitchcock	Wisconsin Medical	Sharp	NARP	Fraser	Austin	Northern	Barwon	Albany	Canterbury	Auckland	TOTAL
Senior managers/leaders	4	1	2	2	0	1	1	0	2	0	2	2	17
Dedicated ACP staff	2	3	3	3	0	1	4	1	3	0	3	1	27
Physicians	2	2	1	2	1	2	3	2	2	4	3	0	21
Nurses	3	2	5	1	8	6	0	0	1	3	5	3	37
Social workers	4	0	5	2	2	1	0	0	1	0	1	1	17
Other	10	6	1	2	1	5	4	0	2	2	3	2	38
TOTAL	25	14	17	12	12	16	12	3	12	9	16	9	157
Individual	19	1	7	12	12	16	12	1	6	9	10	7	112
Group	3	5	3	0	0	0	0	1	2	0	3	1	18

Respondents sometimes filled more than one role. In these cases, we selected the primary role. For example, physicians with a full time clinical position are categorized as physicians even if they are an ACP lead or hold other leadership roles

The category of physicians includes hospital physicians (including palliative care physicians, geriatricians and other specialists) and general practitioners

Dedicated ACP staff are those whose positions are exclusively or predominantly ACP-related

Other includes spiritual care advisors, volunteers, care home staff, speech therapists and occupational therapists

### Data analysis and reporting

Audio-recordings were listened back to in full as soon as possible after the interview and a comprehensive written summary produced. This was entirely descriptive and data elements were included in the same order as the original interview. Time-stamps allowed easy reference back to the audio-recording and potential quotes were included verbatim. Recordings were listened to at least once, but longer and more complex interviews were listened to on two or three occasions, with comprehensive summaries written simultaneously. This process of ‘data reduction’ is appropriate for analyzing large volumes of interview data in thematic analysis and supports the comprehensive and systematic handling of data in analysis [17]. Data management thus also involved several stages, allowing for considerable familiarization. Analytic notes were also taken. Data were then analyzed thematically using NVivo software [17]. The theoretical orientation employed was pragmatic [20]. Given the requirements of fieldwork, interviews and analysis were conducted primarily by a senior researcher and qualitative specialist (JD). A second senior researcher (MK) read a sample of interview summaries, commented on coding frames and provided regular critical input into evolving and final analyses, with any differences resolved through discussion and consensus. Feedback on coding frames and evolving analyses was also obtained from a project advisory group. Descriptive analyses reported in this paper were checked for accuracy by the key contacts in each system; no substantive changes were proposed. Quotes are identified throughout by country and professional role (with these differing slightly from the categories in Table 2, to provide appropriate context while protecting respondent anonymity.)

## Results

Overall, we found many similar experiences and challenges across the twelve healthcare organizations. These are described these from multiple perspectives and with examples from a wide range of settings and contexts, with differences between organizations highlighted as and where relevant. Leadership for ACP support emerged as a key theme and findings on this are described in the first section. The second section describes perceived opportunities and barriers for involving specific types of health and care staff, covering physicians, nurses, social workers, care home staff, spiritual care advisors and volunteers. The third section then describes different models employed by professionals for delivering ACP support, particularly time-intensive ACP conversations, within the context of their existing roles. The implications of these findings are then drawn out in the discussion and conclusion.

### Leadership for ACP support

All of the organizations placed considerable emphasis on leadership, both strategic and day-to-day, for providing ACP support. The exact way this was provided varied between organizations, reflecting their size, structure, stage of development and individual factors. The range of leaders for ACP support provision included senior managers, ACP leads, dedicated ACP facilitators, physician champions and others.

#### Senior managers

Senior managers helped to sustain ACP as a strategic organizational priority, supported those leading ACP support day-to-day and provided strategic leadership during periods of ACP support-related development and change. How active senior managers were varied, between organizations and over time, although senior commitment was generally considered high. At the time of the research, one senior manager reported spending around two hours a week supporting strategic developments for ACP support, reviewing structures for day-to-day leadership and facilitating pilots to generate information on resource needs. Senior managers discussed organizational commitment to ACP support in terms of improving patient care, sustainability, overall cost-efficiency, concerns about potential legal challenges associated with unwanted care, organizational reputation and sectoral leadership. Senior managers’ personal-level commitments to ACP could also sometimes be important. For example, one Chief Medical Officer in the US attributed his sustained and active commitment to experiences working as an emergency physician and from advocating for his father who had recently died with dementia.

#### ACP leads

All of the organizations had an ACP lead, or someone in a similar role, responsible for day-to-day coordination and development of ACP support provision. In two systems (in Canada and Australia), coordination was led by a full-time academic physician, while in both New Zealand organizations the provision of ACP support was led by quality improvement teams as sustained improvement projects. More commonly, however, organizations had established ACP programmes or departments with a full-time salaried ACP coordinator or programme manager. These had backgrounds that included nursing, social work, health education and spiritual care. They commonly reported to senior managers, with the majority feeling well-supported. In the two systems led by academic physicians, ACP support was more research-driven than management-driven and, as a result, support from senior management was more arms-length. The content of the ACP lead role was wide-ranging but could include the provision or facilitation of face-to-face and other training, mentoring and coaching, providing communications and updates, responding to legal and practice queries, networking to secure staff time, developing processes and resources, managing pilots and quality improvement projects, quality control including review of completed documents and working to ensure that ACP is reflected in wider agendas and programmes.

*‘ACP can go off the radar very quickly if you are not present. I sit on a lot of committees; end of life, goals of care document, clinical deterioration, community health.’* (ACP lead, Australia)

ACP leads also sometimes facilitated ACP conversations, including more complex ones. Community outreach was also often an important aspect of the role and this could sometimes involve managing volunteers. In two US systems, ACP coordinators also helped to deliver group facilitations, either in disease-specific support groups or in the community. These involved facilitated group discussions with, for those that wished, an opportunity afterwards to complete ACP documents or, potentially, be referred for an ACP conversation.

#### Dedicated ACP facilitators

Two Australian and two US systems employed dedicated ACP facilitators, specifically to conduct, and support others to conduct, ACP conversations. These were employed part-time, had nursing or social work backgrounds and were acute-sector funded, reflecting that this is where many of the financial and wider benefits of ACP are likely to be realized. Dedicated facilitators were therefore sometimes based in hospitals, conducting conversations at the inpatient bedside and taking referrals from a small number of hospital clinicians or identifying their own clients by consulting patient notes, attending handover meetings and talking with ward staff. In one of the US systems, dedicated facilitators also worked in the community and in one US system and one Australian system, in primary care settings.

A key part of their role was to advise and coach other staff. However, because dedicated facilitators generally had significantly more time available for an ACP conversation than the busy clinicians they were supporting, some respondents believed they were poorly placed to support staff trying to conduct conversations in more time-constrained circumstances. Others thought that having dedicated facilitators gave the impression that ACP was *‘someone else’s job’* and, additionally, could lead to patients experiencing ACP as unintegrated with the rest of their care.

*‘What they generally do is use that money to put in some management and some facilitators who would actually do the facilitation, and the patient will tend to have a silo experience, separate from the rest of the patient journey.’* (Leader, Australia).

However, having dedicated ACP facilitators was thought by some to demonstrate organizational commitment to ACP. They acted as role models and helped to demonstrate that ACP conversations were feasible and acceptable in a clinical context. Their independence was also valued.

‘*If I’ve introduced bias, referring on brings in a check, a more independent person. Patients can try to please their doctor.’* (Hospital physician, Australia).

However, there was wide agreement that over-reliance on dedicated ACP facilitators to conduct ACP conversations was neither sustainable nor scalable.

‘*It can't just be shoved off to one or two passionate individuals working separately in a hospital when they are treating 50,000 people a year.’* (Hospital physician, Australia).

*‘Ten facilitators for the number of patients we have, you know, that’s not going to be possible.’* (Hospital nurse, Canada)

#### Other leaders

Leadership for ACP support was also provided by a range of other staff. In one organization, a senior staff chaplain founded and coordinated a group of volunteers to provide education about ACP in the community. Physicians also acted as champions, promoting ACP to senior managers and clinical colleagues, for example, by attending meetings, giving talks and contributing to training. In some cases, they were engaged in the development of regional and national ACP policy and were occasionally public figures and educators, writing and speaking on ACP and related issues.

#### The impacts of leadership for ACP support

Leaders provided a range of support to help health and care staff within the organization, and sometimes in adjoining systems, deliver ACP support within their existing roles. This support was aimed widely at physicians, nurses, social workers and others such as spiritual care advisors, care coordinators and care home staff. Training offerings varied. These were often generic rather than profession-specific, although shorter tailored training for physicians was common. Staff frequently had access to face-to-face training lasting between half-a-day and, exceptionally, two or three days. Some organizations had also used quality improvement projects, research studies and pilots to help embed ACP support provision and improve processes. As a result of this activity, staff were thought to be widely aware of ACP and its purpose and knew how to access individual support. In organizations with more established programmes, respondents described ACP as a *‘familiar concept’* and *‘part of the culture’* and identified behaviour changes such as physicians having *‘more realistic conversations*.’ Senior leaders, especially where ACP support was more established, also stated that it was highly unlikely they would *‘turn back’* now. We also found many staff members, from all professional backgrounds, providing ACP support in practice. However, those providing ACP support, particularly facilitated ACP conversations (which was thought to take between 30 and 90 min if completed adequately), remained in a minority overall. Leaders also reported that the lack of professional lead role for ACP support could make it difficult to create ownership and accountability, and to target their support efforts.

*‘[Quality improvement team] usually kick things off then hand them over to a permanent home, but there is no obvious home for this, so it’s staying here right now’* (Dedicated ACP staff, New Zealand)

*‘At the moment, everyone's interested but no one's really accountable. It's all done by everyone's good conscience and their ability to buy in and see the value. It's actually no-one's job to do it.’* (Leader, Australia)

Gains also needed to be actively sustained; in two US systems, a sharp decline in awareness and ACP support activity was noted following earlier losses of, respectively, an ACP lead and a highly engaged senior leader.

### Opportunities and barriers for involving different types of health and care staff

#### Physicians

Physicians were seen as key to legitimizing and embedding ACP support provision. They were broadly considered supportive, particularly intensivists and emergency physicians, who worked *‘at the sharp end’,* alongside palliative care physicians, geriatricians, some GPs and some respiratory and renal specialists, while surgical specialists were thought to be amongst the least supportive*.*

*‘There is no great groundswell of resistance. There are some that are just passive, going along with it, and others are either resisters or, a lot, are champions.’* (Leader, Australia)

Nonetheless, even in organizations where ACP support was more established, only a minority of physicians overall were thought to regularly provide ACP support. Reasons were thought to be time constraints; lack of knowledge, skills and confidence; and concerns related to professional autonomy and patient care. Of these, time constraints were considered the most important barrier.

*‘Typically, physicians say it’s our conversation, it’s about prognosis etc. but we don’t have the time.’* (Leader, Canada)

This was thought, by some, to be a particular challenge for hospital physicians.

*‘To do it well you need time. Busy clinicians don’t have this. I think we always feel a bit rushed.’* (Hospital physician, Australia).

*‘The feedback [from a consultation with physicians across the hospital] was unless you are really hammering away at people to do it, it won't get done. People are just too busy.’* (Hospital physician, Australia).

GPs were often considered natural leads for ACP, although some respondents thought that they too lacked sufficient time.

*‘[GPs] can’t possibly be doing all the things they are meant to or they would be working 22 hours a day.’* (GP, US)

*‘[As a GP] I do as much as I can. I’m not doing enough though.’* (GP, New Zealand)

*‘There is this view that the GP is the most appropriate place but I'm not sure why. Whenever I go to the GP there's a hundred people in the waiting room and you get five minutes with them.’* (Leader, Australia)

Physicians were particularly concerned about the impacts of rushing ACP conversations.

*‘It’s not a quick conversation and they don't want to hurt people by having a quick conversation.’* (Physician leader, Australia)

A second set of barriers related to a lack of necessary knowledge, skills and confidence. This included poor communications skills.

*‘If you are really going to get down to talking about prognosis, about what that illness trajectory is going to look like, they [hospital physicians] actually kind of suck at it. Whether its ego, whether they feel they are already doing it, there is a real lack of insight as to how poorly trained they are to do this.’* (Hospital physician, Canada)

*‘Physicians [hospital physicians and GPs] are terrible at helping people understand their choices.’* (GP, US)

At the same time, when supporting physicians with ACP conversations, it was thought important to recognize and build on physicians’ existing communication skills and competencies.

*‘We say, ‘you do a lot of this already.’ We mustn’t offend people by saying this is new.’* (Hospital physician, Australia)

Indeed, one respondent argued that GPs, in particular, often have good communication skills.

*They will talk to a 16-year old about whether she will have an abortion, talk, you know, with alcoholics. They talk about difficult stuff every day. They deal with suicidal young people, depressed elderly. There is nothing specific about advance care planning that is harder than anything else they do.’* (Physician leader, Australia)

Physicians were also sometimes thought to have poor understanding of the medico-legal aspects of ACP, including medical consent and the extent and nature of physicians’ responsibilities to provide treatment. In relation to the above barriers, poor coverage of both communication skills and end of life care in medical training was identified as an important underlying factor.

*‘We can wait for a new generation of physicians and there is some culture change but we’re still not training them properly.’* (Hospital physician, US).

‘*It does seem extraordinary that people who are dealing with really serious illness do not get too much education around end of life. Most people are currently learning on the job, and if you're learning from someone who does it poorly then that's not good.*’ (Leader, Australia)

Training and other support were widely available in the organizations we visited, although commonly physicians failed to access this or undertook short training activities of relatively limited depth. There was a view, however, that GPs in particular were becoming increasingly aware of their need to develop new knowledge and skills in end of life care and there were examples of ACP training included in well-attended continuing professional education courses.

A final set of barriers involved inter-related concerns around professional autonomy and patient care. Some hospital physicians believed, as a matter of principle, that other professionals should not be having conversations with their patients about prognosis and treatment and were, consequently, reluctant to introduce ACP or refer patients for ACP conversations. In some cases, it was thought that they may have doubted the clinical knowledge and skills of facilitators. Physicians may also sometimes have had concerns that patients were being inappropriately advised against active treatment.

*‘Maybe they think people are being persuaded to forego treatment. Some think you should do everything possible.’* (Hospital physician, Australia).

*‘There’s this idea that the job [of ACP conversations] is to get them to agree to palliative care, but that’s not it at all.’* (Hospital physician, Australia).

There were also related concerns about the potential for the involvement of other professionals to impact negatively on trust in the physician-patient relationship.

*‘It potentially plants doubt that the doctors are doing the right thing for you.’* (GP, Australia).

*‘We don’t tell people. ‘don’t trust your healthcare provider’, we teach them to ask the right questions.’* (ACP lead, US).

However, some hospital physicians were thought to want their patients to have completed ACP prior to coming into their care, often so that they could avoid having to initiate these discussions themselves.

*“They can think that someone else should have done advance care planning so they ‘know what to do.”* (Hospital physician, Australia)

#### Nurses

Nurse practitioners and nurses working in chronic disease management were seen to have a particularly important role in ACP support provision. They commonly saw patients over time, in regular and longer appointments and worked more holistically than physicians, with a focus on prevention and well-being. They were also often embedded in clinical teams, working alongside physicians.

*‘Nurse practitioners generally aren’t ‘in the moment’ practitioners. They are thinking about chronic health management. They are thinking about preventative medicine. That’s their space. They are already embedded in teams.’* (Hospital physician, Australia)

*‘Nurses write better plans than physicians, bigger picture, not just clinical decisions. Physicians just want the guts. What patients take away from this is that there are things they can’t talk about.’* (ACP lead, New Zealand)

Nurse-led ACP conversations were also potentially scalable.

*‘We’ve 20 trained nurses [currently], but we have hundreds.’* (Hospital physician, Canada)

*‘The nurse practitioner role is still expanding in Australia, developing as a profession, while society is starting to think about these things.’* (Physician leader, Australia)

While nurses, particularly clinical nurse specialists, practice nurses, nurse care coordinators and nurse practitioners, constituted a large proportion of those delivering ACP support in the participating organizations, it was still the case that, overall, most nurses did not deliver ACP support. Two barriers were identified; time constraints and concerns about legal aspects.

Lack of time was seen as the most significant barrier. For example, in one US system, trained hospital nurses were asked to commit four hours a month to facilitate ACP conversations. This time was not bought out or protected and, in practice, only about half was realized, with nurses regularly having to be *‘chased’*. Competing pressures were a factor, particularly in busy clinical environments. In one US system, one nurse scheduled ACP conversations outside of her normal working hours, taking time off in lieu, to avoid clashes with the responsive commitments of her work role. Other systems faced similar difficulties in involving nursing staff.

*‘We trained general practice nurses. ACP is chronic disease management but their time is so pressured it doesn’t happen in practice.’* (ACP lead, Australia).

*‘They wanted everyone to do one ACP conversation a week. For lots of staff, it was quite unreasonable.’* (Hospital nurse, Canada).

*‘We might just want people to do a conversation a week, but even that makes managers want to run away.’* (ACP lead, US).

Occasionally, it was thought that nurses could have concerns about legal aspects.

*‘Nurses have historically been told not to sign legal documents as there can be conflicts of interest involved.’* (Community nurse, Australia)

#### Social workers

Many systems had health-based social workers and care coordinators with social work backgrounds. Social workers were considered to have an important role in delivering ACP support, since they have a high level of comfort with legal processes and forms, have good facilitation, advocacy and counselling skills and are able to work efficiently with issues such as family conflict and grief.

*‘We’ve had a couple of social workers who’ve been brilliant. They need to sub-contract out the clinical parts of the discussion, but they are really good at bringing it up and really good at facilitation.’* (Hospital physician, Australia)

*‘It needs to be more exploratory, listening for cues. That’s not so much in a nurse’s scope of practice.’* (Social worker, New Zealand)

However, the constraints of some care settings meant that it was often hard for social workers to fully employ these skills and some respondents thought they had become de-skilled.

*‘We're a busy department, we're also counsellors. It can be hard. What's the priority?’* (Social worker, US).

*‘They don’t do any counselling anymore, they’re actually not really trained in it. They’ve really narrowed their scope.’* (Hospital physician, Canada).

The large number of social workers and social work-trained care coordinators in some systems was also, as with nurses, thought to allow for scalability. In practice, social workers were well-represented amongst those delivering ACP support. However, overall, social workers offering ACP support, particularly facilitated ACP conversations, remained in the minority. There were two main barriers identified; time constraints and limited clinical knowledge.

Lack of time was, again, the main barrier, particularly in acute settings.

*‘I do as much ACP as time allows.’* (Hospital-based social worker, US)

*‘I have to protect my time, if it's going to be more than 20 minutes I'll encourage them to read it and do some on their own, and I'll come back.’* (Hospital-based social worker, US).

Social workers who engaged patients in longer appointments in primary care and community-based settings were those most likely to be able to find time to facilitate ACP conversations and, occasionally, social workers specialized. For example, in the US, a social worker conducted, and coached others to conduct, ACP conversations with people with dementia.

A second barrier to the greater involvement of social workers was that they have more limited clinical knowledge than physicians or nurses.

*‘People will tell you what their condition is and you know immediately what kind of issues there might be, and you can ask appropriate questions and advise them about what to ask their doctor. I send people away to ask their doctor further questions. With a non-clinical background, you're going to be doing that a lot more.’* (Hospital nurse, Australia)

However, others thought ACP was ‘*not as clinical as other work’* and, in the US and Canada, social workers reported successfully using leaflets and decision aids to cover key clinical information.

#### Care home staff

Care homes were widely considered a challenging environment for ACP. Barriers included residents having limited or diminishing capacity, staff with limited formal qualifications, high staff turnover and sometimes weak links to the wider health system. Care homes were thought to commonly produce poor quality plans and make insufficient distinction between ACP and liaison with families concerning best interest decisions. Some systems trained care home staff to conduct ACP conversations, which could raise awareness and potentially increase the amount of ACP undertaken. However, in one Australian organization, an initiative to train care home staff to undertake ACP conversations had resulted in low numbers of completed discussions and poor-quality documentation. Respondents commonly discussed the importance of external clinical input into ACP in care homes.

*‘The most useless ones are where people completing the forms or the people helping them to complete the forms clearly don't have any idea of what the options are or the likely outcomes of those options.’* (Hospital physician, Australia).

*‘With care home residents, it’s good to have a geriatrician come in. They can consider what’s feasible and appropriate.’* (Hospital physician, Australia).

GP caseloads made it difficult for them to undertake full ACP conversations in care homes. However, in Australia, we found examples of practice-based nurses, GP registrars and inreach teams (comprising geriatricians and palliative specialists) incorporating ACP support into their work in care homes.

#### Spiritual care advisors

Spiritual care advisors were found mostly in US organizations. They have counselling skills, flexibility to undertake longer ACP conversations and were relatively numerous in some systems. In one organization in particular, spiritual care advisors facilitated a significant proportion of ACP conversations. However, it was occasionally thought that not all patients were necessarily comfortable undertaking ACP with a spiritual care advisor and the demands of providing ACP support could also impinge on their other roles. Spiritual advisors also sometimes held leadership roles, for example, leading community outreach initiatives or, in one case, being employed as a full-time ACP programme coordinator.

#### Volunteers

Volunteers played an important role in community outreach. They were sometimes faith-based, particularly in the US, or had backgrounds as nurses or social workers. In one US example, links were made between the healthcare organization and a volunteer network for providing education about ACP involving local lawyers, which had pre-existed the healthcare system’s own ACP initiative. In another example, in Canada, a Compassionate Communities ACP initiative had been established with provincial funding. This provided seed grants and training for small hospice societies to run ACP workshops, using trialed and tested curricula and teaching materials and with ongoing support from a regional palliative care organization.

### Models for delivering ACP support

We identified a broad typology of models used by professionals for delivering ACP support, particularly time-intensive ACP conversations; these were referral models, team-based models and incorporation models. Referral models were those where professionals, usually physicians, identified someone they believed would benefit from an ACP conversation and referred or sign-posted them to another professional, outside of their immediate clinical or professional team. Team-based models were similar to referral models but involved referral to someone within the same team. Incorporation models involved facilitating ACP conversations in the course of delivering other care, which often required that conversations be broken down into multiple shorter sessions. Occasionally, these different models were not mutually exclusive; for example, where a hospital physician referred to a GP who then incorporated an ACP conversation into other care. However, each of these models had different implications and presented different challenges.

#### Referral models

Referral models were predominantly, but not exclusively, employed by physicians, with referrals made variously to GPs, dedicated ACP facilitators, social workers, spiritual care advisors, care coordinators and nurses. These could work well where the referral was to a trusted professional with adequate time available to facilitate a conversation. However, such referrals were not always secure. For example, three hospital physicians in New Zealand described referring patients to a GP for an ACP conversation upon discharge.

*‘We start in hospital but it gets completed at the GP’s afterwards.’* (Hospital physician, New Zealand)

They identified the benefits as presenting ACP as an integrated part of care and reconnecting the patient and GP prior to subsequent health crises. However, although in this system there was external funding to support GPs to facilitate ACP conversations, GPs were often time-pressured and it was not known if and how GPs responded. Similarly, in Canadian and US-based systems, there were examples of nurse practitioners introducing ACP during home health visits. They encouraged patients to progress their planning with their families and, where possible, their GPs. However, the degree to which GPs were approached or provided this support was unknown. In three organizations in the US, GPs were pro-actively supported to refer patients to dedicated or other trained facilitators. These referrals were mediated through ACP leads and dedicated ACP staff so were more secure, although uptake by GPs varied.

*‘Primary care physicians feel they are getting more and more tasks dumped on them. Our job is to let them know that, although we need them to open up the conversation, there is another resource that they can refer to.’* (Leader, US).

Sometimes physicians referred patients inappropriately in the absence of a suitable resource; for example, in one Australian case, hospital physicians made inappropriate referrals of patients to a hospital-based inreach service, designed to provide palliative care and ACP support to people living in care homes. Professionals also sometimes introduced ACP, with a view to making a later referral; for example, social workers introduced ACP and the prospect of referral to a trained facilitator in introductory meetings with all new patients in a US geriatric clinic.

Another referral route was through outreach activities such as talks, presentations and group facilitations in community settings. Participants could attend these without obligation. Assistance with the completion of documents on the day was sometimes offered, alongside the possibility of referral to a dedicated or other trained ACP facilitator. Many organizations also provided a telephone number that members of the public could use to self-refer. In practice, while community-based events were popular and increased public understanding, referrals to trained facilitators for full ACP conversations were not common.

#### Team-based models

In team-based models, referrals were more secure and physicians could more readily retain involvement. For example, in New Zealand, the US and Australia there were examples of GPs introducing ACP and referring to a nurse practitioner, care coordinator or dedicated ACP facilitator within their practice with, in one case, the GP regularly coming in for the last 5 min of the meeting to briefly review decisions and agree documentation. These were commonly externally funded positions or otherwise attracted some level of additional funding. In another example, in the US, patients presenting repeatedly or avoidably in the emergency room were referred to a hospital-based Advanced Illness Management (AIM) team, consisting of four specialist nurses (with palliative care and oncology specialisms) and a social worker. The team reviewed existing advance directives and, if needed, introduced and conducted ACP conversations. The team could liaise with the referring consultant, as needed, and if a patient preferred a conversation outside of the hospital, they could be referred on to a dedicated ACP facilitator working in the community.

A key benefit of such models is the ability to offer team-based care, with physicians not facilitating entire ACP conversations but, nonetheless, remaining actively involved. However, some respondents identified the risk of physicians using within-team referrals to avoid difficult discussions with patients.

*‘Physicians thought, “do I have to do this? Oh no, this is complicated. I am just going to step back and refer.” We’ve had to train the nurse to bring physicians in as needed. It’s not something ‘someone else’ should do. It should be shared for quality of care.’* (Hospital physician, Canada)

Both referral and team-based models were seen as more cost-effective than entirely physician-led ACP support in that they allow for the longer parts of ACP conversations to be conducted by an appropriate but less costly professional.

*‘Nurses and social workers are so efficient. Physicians don’t need to be there for everything.’* (GP, US).

#### Incorporation models

Staff funded to facilitate ACP conversations included dedicated ACP facilitators and some externally-funded practice staff, while others such as care coordinators and Advanced Illness Teams were funded to provide a range of care that centrally included introducing and having ACP conversations. More commonly, however, health and care professionals did not have any protected time to provide ACP support and, if they could not refer on to another professional, needed to incorporate the provision of ACP support into their existing roles. Attempts to do this generally involved breaking the conversation down into shorter sessions. GPs commonly reported doing this to deliver ACP support.

*‘In my practice, I wrap it up in other consultations. I don’t do it all at one time.’* (GP, US)

In Australia, one GP introduced ACP when drawing up chronic disease management plans, following this up with a conversation undertaken during a standard 15-min consultation (and sometimes a further consultation, if needed). Similarly, in New Zealand, a GP incorporated ACP into annual planning sessions for patients with complex conditions. He explained how only ten minutes were devoted to ACP, but the intention was for this to accumulate, with patients encouraged to progress their planning in between. Nurses and social workers also commonly used this model. For example, professionals conducting home visits including disease-specific specialist nurses in New Zealand, social workers in a US-based home health programme, a palliative care nurse in Australia and care coordinators in the US and Australia conducted ACP conversations incrementally over the course of a series of home visits. In Australia, a nurse manager in a GP practice spent half the week visiting five care homes, during which she undertook short ACP conversations with residents as part of general care, developing them over time. In Canada, heart failure nurses and dialysis nurses incorporated ACP into regular clinic appointments. A potential benefit of breaking the conversation down in this way was that patients had a chance to reflect and have discussions with family members between sessions. It could work well where there was regular and relatively frequent contact with a trusted professional and time was not unduly pressured (e.g. during some types of home visit). However, respondents pointed to the risk of the ACP conversation getting squeezed or of the process becoming inconsistent, fragmented and *‘gappy*.’

In other cases, professionals managed to facilitate ACP conversations within their existing roles by reducing their scope and/or depth. For example, in one US hospital, social workers conducted ACP conversations in regular outpatient clinics and, in Canada, social workers conducted ACP conversations in pre-dialysis clinics. These were busy, however, limiting how much could be covered. The importance of physician input for nurses and social workers providing ACP support in this way was also repeatedly emphasized.

*‘A physician needs to be involved; that’s hard for us in our setting’* (Community nurse, US).

*‘Where the problems are, [patients] often don’t have a good understanding of their illness trajectory. And that’s not the nurse’s role to explain that. The nurses actually can’t. They can help identify where the knowledge gaps are and send them back to the physician.’* (Hospital physician, Canada).

## Discussion

The healthcare organizations in this study were selected for their diversity and varyied, for example, in terms of their size and in the level and type of resources available to them for the development of ACP support. However, in the absence of significant new funding or a professional group with responsibility for leading ACP support, all of the organizations in our study all placed an emphasis on intensive organizational leadership for promoting and sustaining widespread changes in practice. This included strategic leadership from senior managers to maintain ACP as an organizational priority and provide support for strategic development and change. It also, importantly, included intensive day-to-day leadership and coordination from ACP leads. There were local variations in the qualifications, seniority, scope and specific responsibilities of this role. However, in all cases, the purpose was to create and sustain high awareness of ACP, ensure that heath and care staff across the organization were adequately trained and supported, to motivate staff and create accountability, to develop and maintain ACP-related processes, to help maintain agreed standards and to represent the organization in the community and elsewhere on ACP-related issues. The investments in leadership made by organizations were justified and under-pinned by clearly articulated organizational rationales relating to patient care, sustainability, cost-effectiveness, possible legal challenge, reputational benefit and sectoral leadership. Senior managers and ACP leads often worked closely and evidence suggested that organizational awareness and provision of ACP support was significantly increased as a result and that it slipped back when either strategic or day-to-day leadership was absent. Other important leadership for ACP support included support from physician champions, who had a particular role in promoting ACP support to physician and other clinical colleagues. In many organizations, community outreach and public engagement, undertaken as part of a ‘whole system’ approach, was also considered key, as a way of raising public awareness, stimulating interest and demand and creating accountability directly to users and local communities.

While there were various barriers to involving wider health and care staff in the provision of ACP support, there was not thought to be significant resistance to the provision of ACP support in any organization and we heard of numerous professionals, across a wide range of professional groups, delivering ACP support to patients and the wider public. However, professionals providing ACP support, particularly the facilitation of ACP conversations, remained in the minority. From the perspective of leaders, the lack of a clear professional lead group made it difficult to create accountability or target support. For wider health and care staff, the most significant barrier was a lack of protected time, particularly for ACP conversations. However, even introducing ACP could be difficult, especially in busy acute environments or in 15-min general practice appointments. Longer appointments such as annual chronic care management reviews, nurse appointments or some types of home visit presented more opportunity. However, it was commonly considered unrealistic to expect most health and care staff to find the time needed to facilitate ACP conversations routinely within their existing roles. Within organizations, this resulted in variability in the reach, scope and depth of facilitated ACP conversations and also in what one respondent referred to as a *‘significant gap between introducing it and doing it’.* Lack of time is not a novel barrier; the *‘pressure of competing demands’*, for example, is identified as one of the main barriers to implementation in a systematic review of ACP intervention studies [10], while finding time to have adequate conversations is identified as a key barrier to implementation of the RESPECT ACP initiative currently being developed in the UK [21].

Time constraints, however, were not the only barrier. Physician leadership and involvement in the provision of ACP support was universally considered vital; to legitimize the role of ACP in clinical care, to provide clinical input into wider conversations about values and care preferences, to ensure that ACP is well-integrated with day-to-day clinical care and to help ensure the effectiveness of ACP processes. However, physicians experienced the widest range of barriers and concerns. As well as lack of time, physicians sometimes had insufficient communication skills for more values-based discussions, a factor that has been previously identified in the literature as an important barrier in the provision of ACP support and in shared decision-making more generally [22]. Perhaps notably, however, physicians believing that patients would be reluctant to engage in these conversations was not identified as a barrier [9, 10]. This possibly reflects the level to which ACP conversations were normalized within these organizations and the, sometimes extensive, organizational experience gained of patients, members of the public and families finding ACP support acceptable. A barrier we found that is less commonly identified in the existing literature involved physicians having misunderstandings about medico-legal issues, including around the role of ACP in processes of medical consent and the extent of physicians’ responsibilities to treat. Nurses sometimes also had concerns about legal forms, while social workers were considered to be more confident with legal documents and processes. Physicians were also thought to tend towards having conversations of limited scope, late in the disease trajectory. Other concerns arose for some physicians where other professionals initiated and facilitated ACP conversations with their patients. These encompassed potential concerns about facilitators’ clinical skills and knowledge, the possibility that patients may be being persuaded to inappropriately forego treatments, professional autonomy with regard to discussions about prognosis and goals of care and the potential for negative effects on trust in the patient-physician relationship. While the majority of physicians were thought to be supportive of ACP in principle, it also remained the case that most physicians were not engaged actively in the provision of ACP support and the barriers and concerns identified were frequently considered important implementation challenges. One physician and leader in the US noted that *‘physicians have the power to undo everything that has been achieved’,* a viewpoint that is also reflected in the literature about the role of physicians in promoting innovation and practice change more widely [23]. On one hand, these challenges suggest the need for a supportive and sustained approach that works with physicians’ concerns with an emphasis on physician champions and educators. However, there is a need to balance this with the competing concern, also reflected in the existing literature [9, 11, 24], that if ACP becomes predominantly the responsibility of physicians it may become what one physician in our study described as *‘just another treatment decision,’* limited in scope, occurring late in the illness trajectory and with insufficient recognition given to potential risks and burdens.

Models for delivering ACP support were driven primarily by the resource-intensiveness of facilitating ACP conversations, with few staff able to routinely deliver full ACP conversations within their roles. The widespread employment of dedicated ACP facilitators was not considered scalable or sustainable, nor necessarily desirable as it was commonly thought that over-reliance on dedicated staff could lead to lead to poorly integrated care. In one US organization, uniquely, a group of spiritual care advisors were able to find substantial time in their roles to facilitate ACP conversations, although this sometimes impinged on their other responsibilities. In other cases, external sources of funding were sometimes available. For example, in one organization in New Zealand, there was separate District Health Board incentive funding for GPs, usually working with other practice staff, to facilitate ACP conversations and complete plans. This was associated with increased levels of ACP [25]. In other organizations, separate funding streams, including for care coordination and reducing the risk of hospital admission, helped to fund professionals’ time to conduct ACP conversations with relevant target groups. Furthermore, during our fieldwork, the Centers for Medicare and Medicaid Services (CMS) in the US introduced the potential for including ACP conversations in Medicare wellness exams (for people aged 65 and over) as well as ACP-specific Medicare billing codes for an ACP conversation of 30 min and an additional 30 min (for people of any age). Although the level of funding is modest, this development represents the first funding at scale of these longer conversations, with the result that around 575,000 Medicare beneficiaries received an ACP conversation in 2016, twice as many as anticipated by the American Medical Association [26].

More commonly, however, health and care professionals had to find ways of incorporating the provision of ACP support into their existing roles with no additional resource. One model, most commonly used by physicians, was the referral model, which involved introducing ACP and then referring on to other staff for a longer ACP conversation. A number of organizations actively encouraged physicians to use this approach, focusing efforts on encouraging and supporting them to introduce ACP. This could work well where there was a trusted professional with protected time for facilitating ACP conversations to refer on to. However, many referrals appeared insecure, for example, in the case of referrals to GPs who themselves may lack the time to conduct full ACP conversations. Incorporation models were those where ACP support was provided by a single professional alongside and as part of other care. Because of time pressures, this generally involved breaking the conversation down into multiple shorter sessions. If appropriately systematized so that the evolving conversation did not become too fragmented, the merits of this approach included it being easier to incorporate into routine appointments and that it allowed patients and families to reflect and develop their preferences over time. However, this approach could mean that ACP conversations got squeezed out or became too fragmented and, in much shorter sessions, it is possible that facilitators and patients may have felt less able to prompt or raise more complex questions and concerns. For nurses, social workers, care staff and others, the use of an incorporation model was also often associated with insufficient physician input.

Team-based models potentially allowed nurses, social workers and others, to undertake the most time-consuming aspects of providing ACP support, but with physicians retaining active involvement. This shared approach was thought to be the most cost-effective as well as supportive of good quality care. It was also thought to be a good fit with new models of care such as Patient-Centered Medical Homes in the United States and Australia [27] or multi-specialty community providers or primary care homes in England [28, 29]. However, even where facilitation of conversations could be somewhat absorbed into other care, there often remained a need for some additional resource. As noted by one leader in New Zealand, *‘It takes time, but lots of things to do with chronic illness take time.’* Finally, it is worth noting that the challenges of providing ACP in care homes could mean that care home residents have inequitable access to ACP support. Strengthening links with external healthcare providers able to assist in the delivery of ACP support is, therefore, also important [30].

## Conclusion

The emphasis on ACP in national policy is important but not sufficient, on its own, to ensure widespread provision of ACP support in health and care settings locally. This research highlights the need, in the absence of significant new funding or a professional lead role, for intensive and committed organizational leadership, both at a strategic and day-to-day level. The intractability of time constraints, however, remains a significant challenge, particularly for facilitating ACP conversations and, while there may be some scope for absorbing ACP support into existing health and care provision, there is likely to remain a need for some new resource. Physician leadership and involvement are key, and approaches to developing ACP need to work with physicians’ concerns while, at the same time, balancing this against the risk of ACP becoming entirely physician-led, with ACP limited in scope and occurring late in the illness trajectory. Team-based frameworks embedded in evolving models of care for chronic illness and end of life are likely to help to achieve this balance, manage costs and maximize quality of care. The importance of ensuring strong links with external healthcare providers in care homes is emphasized for equity of care. While the full consideration of transferability issues is beyond the scope of this study, the consistency of findings across the four countries provides some confidence that lessons drawn may have relevance in a range of socio-economically similar countries.

## Abbreviations

ACP: Advance care planning
GP: General practitioner
US: United States
